# Serum sclerostin is associated with recurrent kidney stone formation independent of hypercalciuria

**DOI:** 10.1093/ckj/sfad256

**Published:** 2023-11-01

**Authors:** Daniel Rodríguez, Ekaterina Gurevich, Soroush Mohammadi Jouabadi, Eva Maria Pastor Arroyo, Alexander Ritter, Sandrine Estoppey Younes, Carsten A Wagner, Pedro Henrique Imenez Silva, Harald Seeger, Nilufar Mohebbi

**Affiliations:** Division of Nephrology, University Hospital Zurich, Zurich, Switzerland; Division of Nephrology, University Hospital Zurich, Zurich, Switzerland; Department of Internal Medicine , Division of Vascular Medicine and Pharmacology, Erasmus Medical Center, University Medical Center Rotterdam, the Netherlands; Institute of Physiology, University of Zurich, Zurich, Switzerland; Division of Nephrology, University Hospital Zurich, Zurich, Switzerland; Institute of Social and Preventive Medicine (IUMSP), University of Lausanne, Switzerland; Institute of Physiology, University of Zurich, Zurich, Switzerland; Department of Internal Medicine, Division of Nephrology and Transplantation, Erasmus Medical Center, University Medical Center Rotterdam, the Netherlands; Division of Nephrology, University Hospital Zurich, Zurich, Switzerland; Institute of Physiology, University of Zurich, Zurich, Switzerland; Division of Nephrology, University Hospital Zurich, Zurich, Switzerland; Institute of Physiology, University of Zurich, Zurich, Switzerland

**Keywords:** calcium, FGF23, hypercalciuria, nephrolithiasis, phosphate, urolithiasis

## Abstract

**Background:**

Kidney stones are frequent in industrialized countries with a lifetime risk of 10 to 15%. A high percentage of individuals experience recurrence. Calcium-containing stones account for more than 80% of kidney stones. Diet, environmental factors, behavior, and genetic variants contribute to the development of kidney stones. Osteocytes excrete the 21 kDa glycoprotein sclerostin, which inhibits bone formation by osteoblasts. Animal data suggests that sclerostin might directly or indirectly regulate calcium excretion via the kidney. As hypercalciuria is one of the most relevant risk factors for kidney stones, sclerostin might possess pathogenic relevance in nephrolithiasis.

**Methods:**

We performed a prospective cross-sectional observational controlled study in 150 recurrent kidney stone formers (rKSF) to analyse the association of sclerostin with known stone risk factors and important modulators of calcium-phosphate metabolism. Serum sclerostin levels were determined at the first visit. As controls, we used 388 non-stone formers from a large Swiss epidemiological cohort.

**Results:**

Sclerostin was mildly increased in rKSF in comparison to controls. This finding was more pronounced in women compared to men. Logistic regression indicated an association of serum sclerostin with rKSF status. In hypercalciuric individuals, sclerostin levels were not different from normocalciuric patients. In Spearman correlation analysis we found a positive correlation between sclerostin, age, and BMI and a negative correlation with eGFR. There was a weak correlation with iPTH and intact FGF 23. In contrast, serum sclerostin levels were not associated with 25-OH Vitamin D3, 1,25-dihydroxy-Vitamin D3, urinary calcium and phosphate or other urinary lithogenic risk factors.

**Conclusion:**

This is the first prospective controlled study investigating serum sclerostin in rKSF. Sclerostin levels were increased in rKSF independent of hypercalciuria and significantly associated with the status as rKSF. It appears that mechanisms other than hypercalciuria may be involved and thus further studies are required to elucidate underlying pathways.

KEY LEARNING POINTS **What was known**:

- A dysregulation of calcium metabolism is postulated in nephrolithiasis and hypercalciuria is a risk factor for calcium kidney stones.
- Sclerostin is secreted by osteocytes and has effects on bone turnover and calcium metabolism. Deletion of sclerostin in mice reduces calciuria.
- The role of sclerostin in nephrolithiasis has not been addressed.

**This study adds**:

- This study demonstrates that serum sclerostin is associated with the formation of kidney stones in recurrent kidney stone formers. The association was independent of parameters known to influence serum sclerostin levels
- Sclerostin was not associated with calciuria.
- Sclerostin might therefore have a role in kidney stone formation independent of calciuria.

**Potential impact**:

- Studies based on our findings may lead to a better understanding of the pathophysiology of kidney stone formation and the role of sclerostin in this process.
- This could result in new preventive or therapeutic strategies for nephrolithiasis in the future.

## INTRODUCTION

Kidney stones are very frequent in industrialized countries. The lifetime risk is roughly 10 to 15% in these countries [1], with men more often affected than women. A considerable percentage of patients experience recurrent kidney stones with a relapse rate of 35–50% within 5–10 years complicated by an increased risk of coronary heart disease, developing chronic kidney disease and finally end-stage renal disease in the long term [2–4].

Kidney stones are composed of inorganic and organic components. Approximately 80% of patients have calcium-containing stones. Many factors predispose or contribute to the development of kidney stones, including genetic variants, diet, environmental factors, and behaviour [5]. Among all factors, abnormal urinary pH, hypocitraturia, and increased calcium excretion are most frequently found in calcium stone formers and seem to play a major role in the pathogenesis of stone formation [6–10]. A significant percentage of patients with calcium nephrolithiasis and normal parathyroid function also display hypophosphatemia and reduced renal phosphate reabsorption, i.e. a renal phosphate leak with resulting hyperphosphaturia [11–13].

The current view is that calcium stone formers have one or several derangements or imbalances in calcium-phosphate metabolism. Sclerostin, a 213 amino acid (aa) glycoprotein containing a 23 aa signal peptide is encoded by the *SOST* gene and secreted by osteocytes. It inhibits bone formation by osteoblasts via blockade of WNT/β-catenin signalling [14–17]. Deficiency of sclerostin causes van Buchem disease and sclerosteosis, both rare sclerosing bone disorders [17]. There is an increased demand for calcium in sclerosteotic bone disease. This indicates that sclerostin is involved in calcium metabolism. Formally, the absence of sclerostin might either result in increased calcium absorption via the gut or decreased renal excretion. Evidence from a murine sclerostin knockout model suggests that this molecule might regulate calcium reabsorption in the kidney via directly or indirectly modifying the synthesis of 1,25-dihydroxyvitamin D3. Sclerostin knockout mice displayed decreased urinary calcium excretion and increased levels of 1,25-dihydroxyvitamin D3 (calcitriol), yet PTH levels were unchanged [18]. Consequently, increased levels of sclerostin might cause hypercalciuria. The effects of sclerostin on bone turnover and calcium metabolism suggest a potential role of this molecule also in other disorders where bone and calcium homeostasis may be disarranged, such as nephrolithiasis. Interestingly, in humans, sclerostin mRNA is expressed in several tissues, with high levels in the kidney whereas sclerostin protein has only been identified in osteocytes [19, 20]. Even though a dysregulation of calcium phosphate metabolism is postulated in idiopathic calcium stone formers, no prospective or controlled studies have been performed yet on the potential role of sclerostin in nephrolithiasis.

FGF23 is crucial for phosphate homeostasis under physiological and pathophysiological conditions such as X-linked hypophosphatemic rickets or tumor induced osteomalacia. FGF23 is probably the most important regulator of serum phosphate and calcitriol levels in addition to parathyroid hormone (PTH) produced by the parathyroid gland [21–25]. FGF23 is synthesized by osteocytes and osteoblasts and secreted in response to elevated phosphate, PTH, or calcitriol levels. It binds to the FGF receptor (FGFR)/Klotho complex and acts as a phosphaturic hormone by reducing the expression of both sodium dependent phosphate cotransporters, namely NaPi-IIa and NaPi-IIc in renal proximal tubule cells [26, 27]. In chronic kidney disease (CKD) patients, FGF23 is involved in CKD-related mineral and bone disorder (CKD-MBD) and has been suggested to be a cardiovascular risk factor [28]. Little is known about FGF23 levels in patients with calcium nephrolithiasis [11]. Only a few studies have been published with conflicting results on the potential role of FGF23 in the pathogenesis of calcium nephrolithiasis [11, 29–31]. Thus, the role of FGF23 in nephrolithiasis is still not well defined.

Given the large number of kidney stone patients worldwide, a better understanding of the pathogenesis of nephrolithiasis may provide a foundation for the design of more individualized and specifically targeted therapeutics for this patient cohort. The aim of this study was to assess if sclerostin or FGF23 are associated with derangements of mineral metabolism or other metabolic or demographic factors in our cohort of recurrent kidney stone formers (rKFs).

## MATERIALS AND METHODS

### Patients and study design

This study is a prospective cross-sectional observational study including 104 male and 46 female patients (18< age <70 years old) which were referred to our stone clinic for recurrent kidney stone disease and signed a written consent. There were no exclusion criteria. The study was approved by the local ethics committee (KEK Zürich 2011–0448). Patients were enrolled between 1 January 2012 and 12 May 2015 and serum and 24 h urine was collected from all patients at the baseline visit.

As controls, we used non-stone forming patients from the SKIPOGH (Swiss Kidney Project on Genes in Hypertension) cohort. SKIPOGH is a longitudinal family-based study, following the EPOGH (European Project on Genes in Hypertension) protocol [32]. The primary aim of the SKIPOGH cohort study is to explore the role of genes, epigenetic modifications and kidney hemodynamics in blood pressure (BP) regulation and kidney function in the general population. From December 2009 to March 2013, randomly selected participants of the general population were recruited in two regions (Berne *n* = 287 and Geneva 427) and one city (Lausanne *n* = 417) of Switzerland. Inclusion criteria were (i) having a minimum age of 18 years; (ii) being of European ancestry; (ii) having ≥1 and ideally 3 first-degree family members willing to participate; and (iv) providing written informed consent. Baseline examination was conducted between 2009 and 2013. At baseline, data on renal function, cardiovascular and metabolic risk factors as well as on the prevalence of kidney and cardiovascular diseases (CVD) including history of kidney stones were documented. Of course, individuals with kidney stones that had never become clinically symptomatic or radiographically apparent could not be excluded. Random blood and urine samples were also collected at baseline. The study was approved by the ethical committees at each University Hospital before recruitment started. Data analysis from this control group was performed in a retrospective fashion, even though patients were enrolled and data was collected prospectively.

### Biochemical analyses

For sclerostin determination, blood samples were centrifuged for 10 minutes at 3000 rpm and supernatants stored at −80°C until analysis. Serum sclerostin levels were assessed using a highly sensitive second generation ELISA kit (TECOmedical AG, Sissach, Switzerland), according to the instructions specified by the manufacturer [33]. Sclerostin values are reported in pg/ml. The intra- and inter-assay coefficient of variation of this ELISA has been described to be 3.1% and 3.5%. Intact FGF23 levels were determined by enzyme-linked immunosorbent assay (ELISA) according to the manufacturer's protocols (Immutopics International, San Clemente, CA, USA) and given in pg/ml. Intact PTH (iPTH) was measured using an electrochemiluminescence immunoassay (ECLIA) from Roche (Roche Diagnostics GmbH, Mannheim, Germany). 25-OH Vitamin D3 was determined in rKSF by LC-MS/MS (reagents from Chromsystems, Graefelfing, Germany) and in patients from the SKIPOGH cohort using a chemoluminescence assay (LIAISON® 25 OH Vitamin D TOTAL Assay, Diasorin, Saluggia, Italy). 1,25-dihydroxyvitamin D3 was determined in rKSF using a chemoluminescence assay (LIAISON® XL 1,25 Dihydroxyvitamin D, Diasorin, Saluggia, Italy) and in SKIPOGH patients using an ELISA (Immundiagnostik AG, Bensheim, Germany). Blood and urine chemistry parameters were measured using standard laboratory techniques. eGFR was calculated according to the CKD-EPI formula.

### Definitions

Hypercalciuria was defined as urinary calcium excretion >6.25 mmol/day for female and >7.5 mmol/day for male subjects. A stone was considered to be pure if it consisted of ≥95% of a single component. Calcium kidney stones were defined to contain ≥50% of calcium. Stone composition was defined according to Mayo clinic and EAU definitions [34, 35]. Stones containing any struvite were positioned in the struvite group, stones containing any uric acid were placed in the uric acid group, and stones containing any brushite were placed in the brushite group. Stones were classified as calcium oxalate if they contained >50% of CaOx with or without any apatite, stones were classified as apatite if they contained >50% of apatite with or without any CaOx. Patients with stones of unknown composition were placed in the unknown group.

### Statistical analyses

Statistical analyses were performed using Graphpad Prism Version 5 (GraphPad Software, Inc., Boston, Massachusettes, USA), SPSS Version 20.0 statistical package (SPSS Inc., USA) and for the logistic regression R software version 4.0.2 (2020–06-22). Data are reported as mean ± SD. For between-group comparisons, Student's unpaired *t*-test for normally distributed variables and Mann–Whitney test for non-normally distributed variables were used. Spearman correlation was used for correlation analysis. Categorical variables were compared using the chi square test.

One patient in the control group had a very low serum calcium level being equal to 0. Assuming an entry error into the database, the patient was excluded from further analyses. One rKSF patient with an exorbitantly high FGF 23 level [30.6-fold higher than the mean FGF23 (pg/ml) value of all patients] was excluded from the correlation analyses because she had comorbid polycythemia vera (PV). PV is a myeloproliferative neoplasm (MPNs), characterized by clonal proliferation of myeloid progenitors with hematopoietic efficiency. Erythroid progenitor cells highly express FGF23 (reviewed in [36]). Out of concern that FGF23 in this patient is not regulated physiologically, we decided to remove the patient from the analyses. To test whether there was a relationship between sclerostin and kidney stone formation, we applied logistic regression analyses. Two analyses were performed, one containing the variable sclerostin as the only independent variable, the second, with additional pre-specified variables, which we chose based on published literature, to adjust for confounding. Those were age, BMI, eGFR, sex, and serum calcium. For better interpretation, we multiplied calcium values with 10 before entering the regression model. Odds ratios are presented with 95% confidence intervals. Moreover, in our pursuit of optimizing covariate balance between individuals who form kidney stones and those who do not, we employed the propensity score methodology. The propensity score signifies the conditional probability of allocation to a specific group, taking into account the observed covariates [37]. We predicted the propensity score values utilizing a multivariable logistic regression technique embedded within the [matchit] R package [38], incorporating potential confounders such as age, sex, BMI, and eGFR. Subsequently, we matched each stone former individual with one non-former (matching ratio 1:2), utilizing the computed propensity score with greedy nearest-neighbor matching pattern. Fig. S1 (see online supplementary material) shows the distribution of matched covariates pre and post matching.

## RESULTS

### Characteristics of study population and controls

A total of 150 recurrent kidney stone formers (rKSFs) and 388 none-stone-forming controls from the SKIPOGH cohort were included in our study. There was no significant difference in age, weight, BMI, or eGFR between male rKSF (*n* = 104) and control male subjects (*n* = 189). Female rKSFs (*n* = 46) had a significantly higher weight and BMI compared to female controls (*n* = 199) (Table 1). The number of stone episodes was not significantly different between male and female rKSF (Table 2). As expected, calcium oxalate was the most frequent stone type and was significantly more common in male compared to female stone formers. Second most common were apatite stones in women and urate stones in men. Apatite kidney stones were significantly more frequent in females. Few patients had brushite stones and one woman had a struvite stone. Stone composition was unknown in 6.7% of patients (Table 2). A total of 53.3% (80) of the stones were pure and 40% (60) were mixed and 63% of male patients had pure stones compared to 33% in women, whereas women more often had mixed stones (57%) compared to men (33%).

**Table 1:** Baseline characteristics (total, men, and women).

	Male (*n* = 293)		Female (*n* = 245)	
Characteristics	rKSF (*n* = 104)	Control (*n* = 189)	rKSF (*n* = 46)	control (*n* = 199)
Age, yr (SD)	46.7 (13.2)	46.8 (17.5)	45.8 (14.7)	49.2 (17.6)
Weight , kg (SD)	81.8 (15.0)	81.5 (13.5)	71.5 (17.8)	63.2 (10.6)**
Body mass index, kg/m^2^ (SD)	26.4 (4.6)	25.9 (4.1)	26.5 (6.3)	23.4 (3.7)**
eGFR, ml/min (SD)	96.5 (17.6)	101.0 (17.4)	95.1 (22.2)	96.4 (18.3)

Data are presented as mean ± SD or percentage of patients. The *P* value refers to differences between men and women. Student's unpaired *t*-test and Mann–Whitney test were used where appropriate. Baseline characteristics of rKSF and control patients according to gender. Data are shown as mean ± SD, *P ≤ 0.05, **P ≤ 0.01, ***P ≤ 0.001.

**Table 2:** Average number of stone episodes and stone composition of rKSF according to sex.

	Male (*n* = 104)	Female (*n* = 46)	*P*
Number of stone episodes mean (SD)	2.9 (3.3)	2.2 (1.3)	0.246
**Stone type**			
Calcium oxalate, *n* (%)	85 (81.7)	25*** (54.3)	0.0005
Apatite, *n* (%)	4 (3.8)	13*** (28.3)	<0.0001
Brushite (%)	4 (3.8)	1 (2.2)	0.5988
Uric acid, *n* (%)	6 (5.8)	1 (2.2)	0.6764
Struvite, *n* (%)	0 (0.0)	1 (2.2)	0.1314
Unknown, *n* (%)	5 (4.8)	5 (10.0)	0.1699

*P ≤ 0.05, **P ≤ 0.01, ***P ≤ 0.001.

A total of 134 out of 150 patients had calcium-containing kidney stones. That is, stones which contained >50% calcium (two of the uric acid stones in males contained >50% calcium). In the 10 patients with unknown stone composition, computed tomography was available in eight patients. Four of the eight had stones with >500 Hounsfield units, which has previously been described to be a predictor of calcium-containing stones [39, 40]. Taken together, approximately 92% of our study population had stones, which mainly consisted of calcium. Yet, in the subgroup analysis of patients with calcium-containing stones, only patients with known stone compositions were included.

Table 3 displays blood and urine chemistry of rKSFs and controls according to sex. Interestingly, iPTH was on average 27% higher in rKSFs compared to controls. The difference was even more pronounced for 1,25-dihydroxyvitamin D3 (57% higher in rKSFs), whereas 25-OH-Vitamin D3 was slightly (17%) but significantly lower in rKSF. In addition, calciuria was almost twice as high in rKSFs compared to controls and 24 h sodium excretion was greatly increased (40%) in patients with kidney stones. rKSFs had a 28% higher urine volume compared to controls. The numbers were similar when we only analysed rKFs with calcium kidney stones (i.e. stones composed of ≥50% calcium) (Table S1, see online supplementary material).

**Table 3:** Blood and urine chemistry of rKSF and controls according to sex.

	Male				Female			
	rKSF (*n* = 104)		Control (*n* = 189)		rKSF (*n* = 46)		Control (*n* = 199)	
Blood parameters	Mean	SD	Mean	SD	Mean	SD	Mean	SD
Creatinine in umol/l	83	14	78***	12	68	14	64	11
Sodium in mmol/l	141	3	137***	2	139	2	137	3
Potassium in mmol/l	3.9	0.4	4.1***	0.4	3.8	0.3	4.1***	0.4
Magnesium in mmol/l	0.83	0.06	0.88***	0.07	0.81	0.07	0.89***	0.06
Bicarbonate in mmol/l	26.9	2.5	27.8**	1.7	25.1	2.5	27.4***	2.1
Uric acid in umol/l	350	79	358	65	260	58	266	63
Urea in mmol/l	5.2	1.5	4.7**	1.4	5.1	1.5	4.2***	1.3
Calcium in mmol/l	2.4	0.1	2.3***	0.1	2.4	0.1	2.3***	0.2
Phosphate in mmol/l	0.94	0.15	1.04***	0.16	0.96	0.17	1.15***	0.17
iPTH in pg/l	50.2	18.5	39.5***	12.8	51.6	23.6	40.3***	15.6
25-(OH)_-_Vitamin D3 in ng/ml	16.3	7.7	19.5**	8.6	18.4	9.3	21.2*	9.8
1.25-(OH)_2-_Vitamin D3 in ng/ml	57.1	19.9	37.6***	13.3	64.3	34.6	38.1***	12.9
Urine parameters	mean	SD	mean	SD	mean	SD	mean	SD
Volume in ml	2033	837	1645***	806	2244	1078	1626***	634
Urinary pH	6.2	0.4	5.6***	0.6	6.3	0.5	5.6***	0.7
Sodium in mmol/d	190.5	80.1	151.0***	64.6	165.7	67.6	111.8***	47.0
Potassium in mmol/d	61.3	24.5	73.8***	23.8	58.8	24.1	59.6	22.6
Chloride in mmol/d	177.6	78.1	149.2**	60.0	150.5	64.7	111.7***	44.7
Calcium in mmol/d	6.1	3.2	4.3***	2.5	5.7	2.9	3.7***	2.1
Magnesium in mmol/d	4.1	1.9	4.5*	0.1	3.6	1.8	3.7	0.1
Phosphate in mmol/d	29.5	11.5	31.9	10.2	24.6	9.5	22.8	7.1
Urea in mmol/d	399	146	409	123	327	122	309	86
Creatinine in mmol/d	15.0	4.1	16.4*	3.9	10.0	2.9	10.5	2.7
Uric acid in mmol/d	3.4	1.3	3.9***	1.1	2.8	0.9	3.0	0.8

*P ≤ 0.05, **P ≤ 0.01, ***P ≤ 0.001

### Serum sclerostin was increased in rKSF

**Figure 1:**
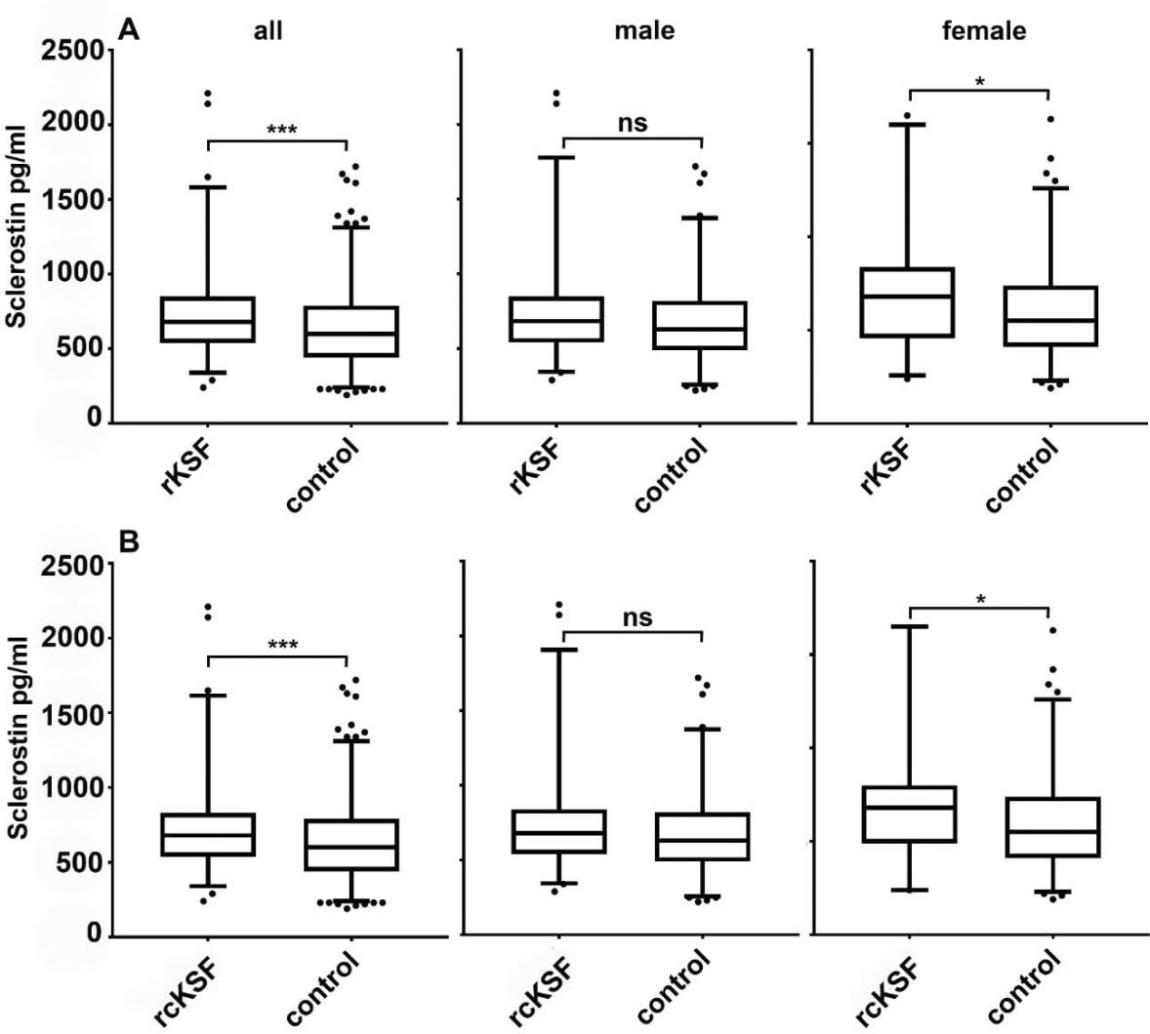
Serum sclerostin (pg/ml) in rKSF compared to control. (**A**) all rKFS compared to controls. (**B**) recurrent *calcium* kidney stone formers rcKSF (defined as calculus with calcium content of ≥50%); the box extends from the 25th to the 75th percentiles, the line at the middle of the box depicts the median, whiskers are drawn down to the 10th percentile and up to the 90th, points below and above the whiskers are drawn as individual points; *P ≤ 0.05, **P ≤ 0.01, ***P ≤ 0.001, ns = *P *> 0.05.

**Figure 2:**
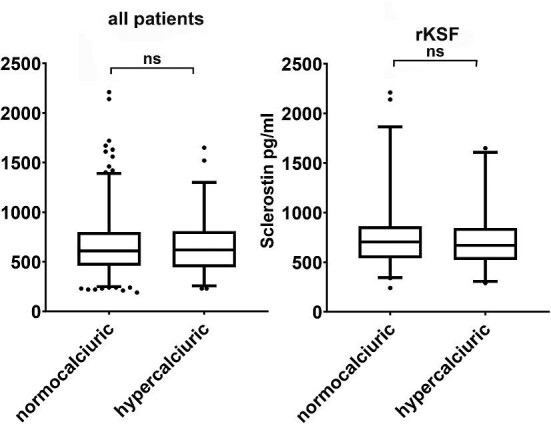
Serum sclerostin (pg/ml) in normocalciuric and hypercalciuric patients. Left: all patients (*n* = 437 normocalciuric, *n* = 101 hypercalciuric), right: rKSF (*n* = 98 normocalciuric, *n* = 52 hypercalciuric); the box extends from the 25th to the 75th percentiles, the line at the middle of the box depicts the median, whiskers are drawn down to the 10th percentile and up to the 90th, points below and above the whiskers are drawn as individual points; *P ≤ 0.05, **P ≤ 0.01, ***P ≤ 0.001, ns = *P *> 0.05.

In rKSFs mean serum sclerostin was significantly higher (17%) compared to controls (740 ± 316 in rKSFs vs 632 ± 263 pg/ml; *P *< 0.001). In male stone formers there was a trend towards higher values, but the difference (11%) was non-significant (754 ± 325 vs 678 ± 269 pg/ml; *P *= 0.061), whereas the difference (20%) in female patients was (707 ± 296 vs 588 ± 250 pg/ml; *P *= 0.011) (Fig. 1A). Findings were similar, when only rKFs with calcium stones were analysed (all patients: 732 ± 316 pg/ml, controls: 632 ± 263 pg/ml; *P *= 0.001) (males: 746 ± 327 pg/ml, male controls 678 ± 269 pg/ml; *P *= 0.122) (females: 701 ± 290, female controls 588 ± 250 pg/ml; *P *= 0.020) (Fig. 1B). Interestingly, in hypercalciuric patients (*n* = 101) serum sclerostin levels were not different compared to normocalciuric individuals (*n* = 436) (646 ± 254 vs 665 ± 289 pg/ml; *P *= 0.792). The same was observed when only hypercalciuric rKSFs (n = 52) and normocalciuric rKSFs (n = 98) were compared (708 ± 268 vs 757 ± 339; *P *= 0.567) (Fig. 2).

We also analysed sclerostin levels according to stone type and sex. Interestingly, in patients with uric acid stones the average sclerostin level was higher than in calcium oxalate, brushite and apatite (Table S4, see online supplementary material). This finding is very interesting considering our results and previous data reporting a higher incidence of uric acid stones in KSF with overweight and sclerostin correlating with fat mass and/or BMI [41, 42]. However, due to the low numbers we refrained from further statistical analyses.

### Sclerostin was associated with age, BMI, eGFR, PTH, and FGF23

In the entire population, serum sclerostin concentration significantly correlated with age (r = 0.47), BMI (r = 0.36), and inversely with eGFR (r = −0.42). There was a weak correlation with iPTH (r = 0.18), FGF23 (r = 0.19), plasma phosphate (r = −0.17) and chloride (r = −0.15). Serum sclerostin did not correlate with serum 25-OH Vitamin D3, 1,25-dihydroxy Vitamin D3, 24-hour urinary calcium or phosphate excretion (Table 4). Correlations were mostly consistent with findings in all patients, when only control patients were analysed (Table S3, see online supplementary material). In control patients, 24 h urine citrate and oxalate values were not available. In rKSFs, sclerostin and 24 h urine citrate excretion were not correlated (Spearman correlation coefficient, R = 0.11, *P *= 0.17). Similarly, there was no significant correlation between sclerostin and 24 h urine oxalate excretion (R = 0.005, *P *= 0.5585).

**Table 4:** Spearman correlation between plasma and urine factors in all patients.

	FGF23	age	BMI	eGFR	Ca	PO4	PTH	25VD	1–25VD	Na	Cl	Bicarbonate	24hUCa	UCa/UCrea	24hUPO4	UPO4/UCrea	24hUNa	UNA/Crea
Sclerostin	.185***	.466***	.359***	−.420***	.085*	−.167***	.178***	−0.03	0.06	.134**	−.154***	0.07	−0.03	−0.03	0.03	−0.03	0.03	−0 003
FGF23		.096*	0.08	−.123**	.205***	0.01	.121**	0.07	0.02	.092*	−.139**	0.00	−0.07	−0.05	0.03	.102*	−0.01	0 042
age			.275***	−.747**	−.107*	−.121**	.400***	−.115**	−.100*	0.07	.099*	0.03	−.114**	0.02	−.101*	.139**	−0.05	.106*
BMI				−.254***	.132***	−.241***	.228***	−.141**	0.05	0.07	−0.08	−0.08	.096*	−0.05	.298***	−0.01	.282***	0 060
eGFR					0.01	.100*	−.295***	0.02	0.08	−0.04	−0.02	0.03	.180***	0.08	.110*	−0.05	0.06	−0 059
Ca						−.180***	0.02	−.089*	.237***	.375***	−.265***	.106*	.237***	.211***	0.06	0.01	.211***	.210***
PO4							−.255***	.175***	−.191***	−0.08	0.07	.091*	−.169***	−0.08	−.091*	.108*	−.189***	−.096*
PTH								−.354***	.108*	.134**	0.01	−0.08	−0.08	−0.04	−0.01	0.03	0.07	.149**
25VD									0.03	−0.07	0.02	0.03	0.08	.113**	0.01	0.07	−0.08	−.069
1–25VD										.118**	−.177***	−.094*	.221***	.208***	0.03	−0.01	.169***	.143**
Na											.221***	.181***	.128**	.129**	0.01	−0.06	.119**	.117**
Cl												−.199***	−.106*	−0.05	−0.07	0.00	−.090*	−.038
Bicarbonate													0.00	−0.04	0.03	−0.04	−.096*	−.163**
24hUCa														.845***	.416***	.211***	.450***	.282***
UCa/UCrea															0.05	.331***	.218***	.390***
24hUPO4																.474***	.485***	.021
UPO4/UCrea																	.081	.245***
24hUNa																		.736***
UNA/Crea																		

Empty cells represent values presented elsewhere in the table. *P ≤ 0.05, **P ≤ 0.01, ***P ≤ 0.001.

Interestingly, in rKSFs sclerostin was not associated with plasma phosphate or PTH. Correlation analyses in rKSF and controls did not show an association of sclerostin with any of the tested urinary variables (Table 4; Tables S2 and S3, see online supplementary material).

### Sclerostin associated with stone formation

To investigate the effect of sclerostin on stone formation we applied logistic regression analyses. Both the unadjusted logistic regression and the analysis corrected for possible confounders (age, sex, BMI, eGFR, serum calcium, and FGF23) showed a highly significant and independent effect of sclerostin on overall stone formation (Table 5).

**Table 5:** TBC. Logistic regression analyses.

Stone former = yes				Stone former = yes		
Predictors	Odds ratio	CI	*P*	Odds ratios	CI	*P*
Sclerostin	3.67	1.91–7.20	**<0.001**	3.84	1.46–1.03	**0.006**
Age				0.97	0.95–0.99	**0.014**
Male sex				2.12	1.26–3.61	**0.004**
BMI				1.05	0.99–1.11	0.096
eGFR				0.98	0.97–1.007	0.21
Serum calcium				3.69	2.82–4.95	**<0.001**
FGF23				1.007	1.002–1.013	**0.016**
(Post-Matching)	Stone former = yes			Stone former = yes		
Predictors	Odds ratio	CI	*P*	Odds ratios	CI	*P*
Sclerostin				3.45	1.24–10.34	**0.02**
Serum calcium				4.60	3.22–6.88	**<0.001**
FGF23				1.009	1.002–1.019	**0.039**

Two logistic regression analyses were performed, one containing the variable sclerostin as the only independent variable (left), the second (right) with additional variables (age, gender, BMI, eGFR, serum calcium) to adjust for confounding factors. The dependent variable was being a stone former. The lower half of the table depicts results post matching for age, sex, BMI, and eGFR. The odds ratio indicates the change in the relative probability of being a stone former when the independent variable increases by one unit (1 ng/ml or 1000 pg/ml for sclerostin; 0.1 mmol/l for calcium). CI, confidence interval. *P*-values bold (significance level < 0.05).

Because the female control group, in particular, was not well balanced, we performed a sensitivity analysis using propensity score matching. The propensity score matching effectively balanced the distribution of age, sex, and BMI variables between individuals who formed kidney stones (*n* = 149) and those who do not (*n* = 149). However, it did not achieve complete balance with respect to eGFR between these two groups [see Materials and Methods and Fig. S1 (see online supplementary material)]. Compared to the unmatched model, the direction and magnitude of the sclerostin effect on stone formation remained consistent (Table 5).

### Serum FGF23 increased in female rKSF

**Figure 3:**
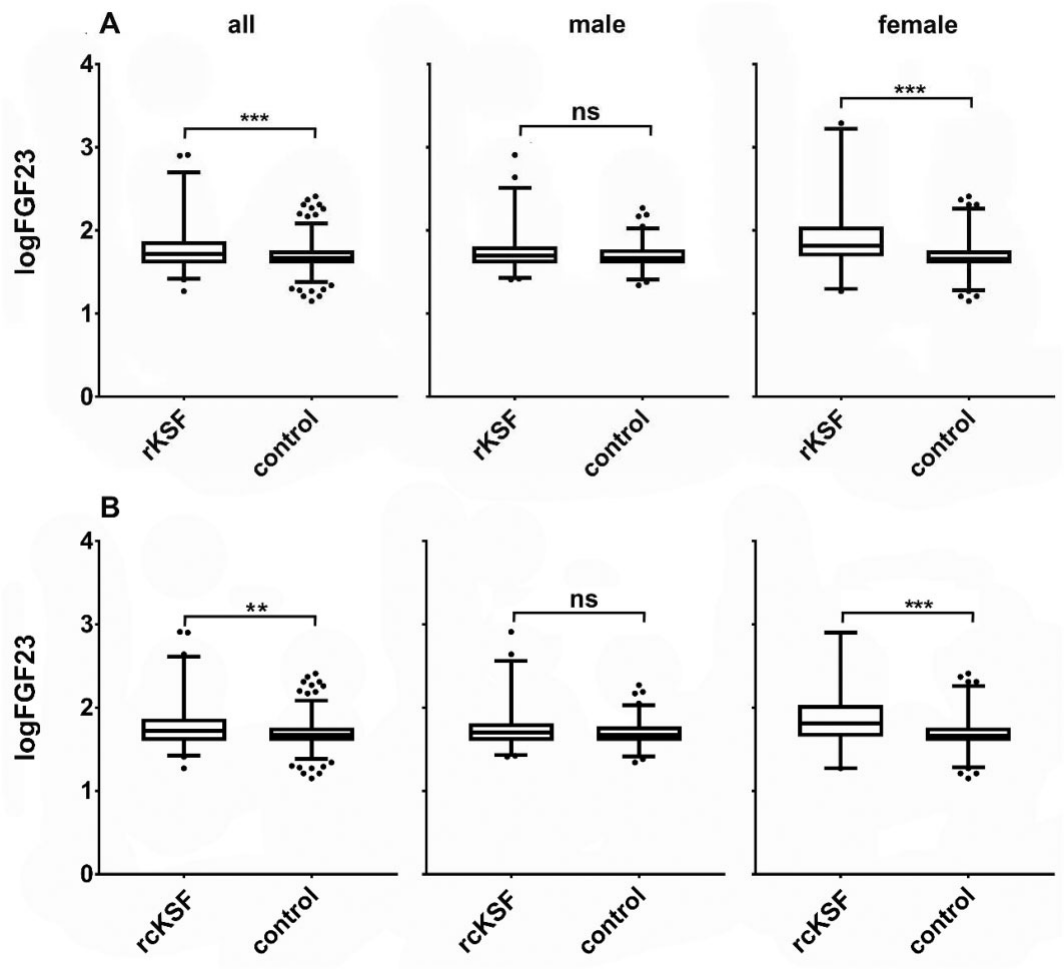
Serum logFGF23 levels (pg/ml) in rKSF compared to controls. (**A**) all rKFS compared to controls. (**B**) recurrent calcium kidney stone formers rcKSF (defined as calculus with calcium content of ≥ 50%); the box extends from the 25th to the 75th percentiles, the line at the middle of the box depicts the median, whiskers are drawn down to the 10th percentile and up to the 90th, points below and above the whiskers are drawn as individual points; *P ≤ 0.05, **P ≤ 0.01, ***P ≤ 0.001, ns = *P *> 0.05.

We observed slightly higher mean logFGF23 concentrations in rKSFs compared to controls (1.8 ± 0.29 vs 1.7 ± 0.17; *P *= 0.001). When rKSFs were split into males and females we only observed a significant difference in female rKSFs vs controls (logFGF23 1.9 ± 0.4 vs 1.7 ± 0.2; *P *< 0.001) but not in male rKSFs (logFGF23 1.7 ± 0.4 vs 1,7 ± 0.2; *P *= 0.369) (Fig. 3A). Similar differences where observed when only recurrent calcium stone formers were analysed (Fig. 3B).

### FGF23 levels correlated with eGFR, serum phosphate, and PTH levels in rKSF

In our cohort of recurrent stone formers and controls, FGF23 serum levels weakly correlated with plasma calcium (r = 0.21), serum sclerostin (r = 0.19), iPTH (r = 0.12), and inversely with plasma chloride (r = −0.14) and eGFR (r = −0.12) (Table 4). In rKSFs we observed a moderate correlation with age (r = 0.33) and eGFR (r = −0.26), plasma bicarbonate (r = −0.25), plasma phosphate (r = 0.17), iPTH (r = 0.17) and plasma sodium (r = −0.16). Interestingly, we did not observe a correlation between plasma FGF23 and 24 h urine phosphate excretion and only a weak correlation with urine phosphate/creatinine ratio (r = 0.17). There was an inverse correlation between FGF23 and 24 h urinary calcium and sodium excretion (r = −0.24 and r = −0.20). We did not observe a correlation between serum FGF23 and 25-OH or 1,25-dihydroxy Vitamin D3 (Table S2, see online supplementary material).

### FGF23 levels in patients with renal phosphate leak

To investigate whether FGF 23 is increased in rKSF with renal phosphate leak we identified patients with decreased serum phosphate (<0.87 mmol/l), normal PTH (<65 ng/l) and increased urinary phosphate excretion (>22.6 mmol/d; *n* = 24). We compared this group to patients with normal phosphate levels in blood and urine (serum phosphate >0.87 mmol/l, normal PTH and urinary phosphate excretion <22.6 mmol/d; *n* = 32). There was no difference in FGF23 levels between these two groups.

## DISCUSSION

The pathophysiology of calcium kidney stone formation is still incompletely understood. Whereas several studies have investigated associations of serum sclerostin with disorders of bone [43–45], vascular disease [46], chronic kidney disease (CKD) [47–50], and mortality [51–55], so far, no prospective or controlled studies are available concerning the role of sclerostin in nephrolithiasis. Menon and others assessed local expression of sclerostin in stone-forming patients with idiopathic hypercalciuria (IH) by immunostaining of undecalcified bone. Sclerostin, but not FGF23 immunoreactivity was increased in IH stone patients with high bone resorption compared to IH subjects with normal bone resorption. This study points to a role of sclerostin in IH; however, only iliac biopsies were investigated and systemic sclerostin levels as well as urinary parameters were not measured [56]. Also, a recent retrospective study from Rodrigues *et al.* reported an association of serum sclerostin with urinary calcium in stone formers [41].

In our prospective controlled study, we investigated sclerostin and FGF23 in rKSFs and controls to test their potential role in recurrent kidney stone formers. Our cohort of rKSF is a well characterized and representative stone cohort including complete blood and 24 h urine analyses and importantly the exact stone composition in 93.3% of the patients investigated. As expected, most of the patients included in the study had calcium-containing kidney stones.

In this cohort, mean sclerostin plasma levels in control patients with negative history for kidney stones was 632 ± 263 pg/ml. The average sclerostin level in our patients was similar to the average sclerostin level determined by McNulty and colleagues in serum from 15 healthy female and 10 healthy male subjects using the same assay as in our study [33]. Interestingly, in recurrent stone formers, sclerostin serum concentrations were significantly increased by 17% (740 ± 316 pg/ml) compared to controls. They were slightly higher in males (754.4 ± 325) than in females (707 ± 296). A more detailed analysis by sex demonstrated that the difference between stone formers and controls was mostly because of elevated sclerostin levels in female rKSFs. Several findings could explain this difference. First, stone composition was significantly different in male and female patients. Men had significantly more CaOx stones while women presented with more apatite stones. The different pathophysiology of these two stone types could thus explain the variances in sclerostin levels. Second, the control group in women was not matching as well as for male patients. Female control patients had a lower BMI compared to stone forming women, which may result in lower sclerostin levels in the control group, as sclerostin has been shown to be positively associated with BMI.

We further compared sclerostin levels in normocalciuric to hypercalciuric patients. Interestingly, no differences were observed, neither in the entire study population (Fig. 2 left), nor among hypercalciuric *versus* normocalciuric rKSFs (Fig. 2 right). This is in line with findings from a recent study by Ramalho and colleagues who observed that in patients with CKD, sclerostin was not associated with urinary calcium excretion [57]. Logistic regression analysis indicated that sclerostin in blood, even when corrected for known and potential confounders (age, sex, BMI, eGFR, serum calcium, and FGF23), was significantly associated with the status as recurrent kidney stone former and stone type, namely uric acid stones Taken together, our results indicate that sclerostin may be involved in kidney stone formation; however, independent of urinary calcium excretion. We may thus speculate that sclerostin is involved in the context of metabolic stone disease and the interrelationship of BMI, uric acid stones, urinary acidification, and potentially sclerostin levels.

In the entire study population, sclerostin blood levels were significantly correlated with serum FGF23, PTH, age, and BMI and a negative correlation was found for eGFR and phosphate. Interestingly there was no correlation between sclerostin and 24 h urinary excretion of calcium or phosphate. In addition, there was no correlation between sclerostin and 25-OH- and 1, 25-dihydroxyvitamin D3 levels in plasma, both important mediators in calcium-phosphate metabolism.

When analysed separately, we did not observe a correlation between sclerostin and phosphate or PTH in rKSFs as we did in the control patients. Whether the latter findings are reproducible and indicate a dysregulation in sclerostin physiology in rKSFs needs to be determined in future studies.

The role of FGF23 in kidney stones formation has not been clarified yet. We show that FGF23 levels in rKSFs as well as in the subpopulation of recurrent calcium KSFs were slightly increased compared to controls. Interestingly, we only detected this difference in female rKSFs but not in males. This could hint to a subtle sex difference in the pathophysiology of kidney stone formation as has been suggested previously for other hormones [58]. An earlier study also found a trend towards higher FGF23 levels in incident stone formers [30]. Interestingly, Ketha and others could not detect differences in FGF23 levels in first time stone formers [59].

In our population of rKSFs we did not notice increased levels of FGF23 in individuals with renal phosphate leak, which confirms findings by Dhayat and co-workers in Swiss kidney stone formers [31] but contradicts an earlier study from an Italian population which demonstrated strongly increased FGF23 in KSF with phosphate leak [11]. The difference to the latter study might be attributable to genetic or dietary differences between the two populations or to the fact that we measured intact FGF23 while Rendina and colleagues reported the C-terminal fragment. Taken together, earlier studies and the results of this investigation do not suggest a major contribution of FGF23 to kidney stone pathophysiology in patients with or without renal phosphate leak even though the issue deserves future scrutiny.

There are few limitations to our study. There were some differences in demographic data in the control group compared to rKSF that may impact the results reported. Sclerostin was measured in our study using a highly sensitive second generation ELISA assay. Delanaye *et al.* compared four different sclerostin assays. There was a high inter-assay variability and a negative correlation between eGFR and sclerostin was found for two of the assays (including the one we used), whereas for the other two assays, there was no correlation. There were also differences in the correlation with other patient and laboratory parameters in the study between assays [60]. Another limitation is that we did not measure sclerostin in bone and sclerostin levels in the serum might not necessarily reflect its activity within bone [56].

Another limitation is that most of the differences in sclerostin but also in FGF23 serum levels were observed in females. Female kidney stone formers had higher weight and BMI compared to controls and as described above, BMI interacts with sclerostin. In addition, these groups may also have had undetected differences that could not be controlled for (i.e. differences in diet or bone metabolism) which could have affected sclerostin and FGF23 serum levels.

In summary, this is the first study prospectively investigating serum sclerostin levels in rKSF. Sclerostin levels were increased in rKSF compared to controls and logistic regression exhibited a statistically significant relationship between serum sclerostin and kidney stone formation. This might indicate a potential role of sclerostin in kidney stone formation, notably, independent of urinary calcium excretion. Sclerostin levels were markedly higher in female stone formers compared to men. In hypercalciuric individuals, sclerostin levels were not different from normocalciuric patients, neither did sclerostin levels correlate with 25-OH Vitamin D, 1, 25OH-Vitamin D or 24-hour urinary calcium excretion, indicating that serum sclerostin might not play a major role in the pathophysiology of hypercalciuria. Our results need to be confirmed in larger stone cohorts to provide evidence if and through which mechanisms sclerostin has an impact on the pathophysiology of recurrent nephrolithiasis.

## Supplementary Material

sfad256_Supplemental_Files

## Data Availability

The data underlying this article will be shared on reasonable request to the corresponding author.
