# Supplementary material for: Serum sclerostin is associated with recurrent kidney stone formation independent of hypercalciuria
Source: Clin Kidney J. 2023 Nov 1;17(1):sfad256. doi: 10.1093/ckj/sfad256 (PMC10768761; doi:10.1093/ckj/sfad256)
Supplement: sfad256_Supplemental_Files [file sfad256_supplemental_files.zip › Supplemental table 1 blood and urine chemistry only calcium stone patients_final.docx]

|  | **male** | | | | **female** | | | | |
| --- | --- | --- | --- | --- | --- | --- | --- | --- | --- |
| **Blood parameters** | **rKSF (n=95)** | | **control (n=189)** | | **rKSF (n=39)** | | **Control (n=199)** | | |
|  | **mean** | **SD** | **mean** | **SD** | **mean** | **SD** | **mean** | **SD** |  |
| Creatinine in umol/l | 82,4 | 14,7 | 78,3* | 12,1 | 67,4 | 11,5 | 64,2 | 11,4 |  |
| Sodium in mmol/l | 140,9 | 2,8 | 138,6*** | 2,3 | 139,5 | 2,3 | 138,6* | 2,5 |  |
| Potassium in mmol/l | 3,9 | 0,4 | 4,1*** | 0,4 | 3,8 | 0,3 | 4,1*** | 0,4 |  |
| Magnesium in mmol/l | 0,83 | 0,06 | 0,88*** | 0,07 | 0,82 | 0,06 | 0,89*** | 0,06 |  |
| Bicarbonate in mmol/l | 27,0 | 2,5 | 27,8** | 1,7 | 25,2 | 2,2 | 27,4*** | 2,1 |  |
| Uric acid in umol/l | 347,0 | 77,0 | 357,9 | 65,1 | 261,3 | 52,9 | 266,2 | 62,8 |  |
| Urea in mmol/l | 5,2 | 1,6 | 4,7* | 1,4 | 4,9 | 1,2 | 4,2*** | 1,3 |  |
| Calcium in mmol/l | 2,4 | 0,1 | 2,3*** | 0,1 | 2,4 | 0,1 | 2,3*** | 0,2 |  |
| Phosphate in mmol/l | 0,94 | 0,15 | 1,04*** | 0,16 | 0,93 | 0,15 | 1,15*** | 0,17 |  |
| iPTH in pg/l | 49,7 | 17,9 | 39,5*** | 12,8 | 51,9 | 21,5 | 40,3*** | 15,6 |  |
| 25-(OH)_-_Vitamin D3 in ng/ml | 16,0 | 7,5 | 19,5*** | 8,6 | 17,7 | 8,5 | 21,2* | 9,8 |  |
| 1,25-(OH)_2-_Vitamin D3 in ng/ml | 56,7 | 19,9 | 37,6*** | 13,3 | 61,4 | 22,9 | 38,1*** | 12,9 |  |
| **Urine parameters** |  | |  | |  | |  | | |
|  | **mean** | **SD** | **mean** | **SD** | **mean** | **SD** | **Mean** | **SD** |  |
| Volume in ml | 2023 | 794 | 1645*** | 807 | 2258 | 1082 | 1626*** | 63 |  |
| Urinary pH | 6,2 | 0,4 | 5,6*** | 0,6 | 6,4 | 0,5 | 5,6*** | 0,7 |  |
| Sodium in mmol/d | 192,7 | 81,2 | 151,0*** | 64,6 | 169,5 | 66,5 | 111,8*** | 47,0 |  |
| Potassium in mmol/d | 61,2 | 24,5 | 73,8*** | 23,8 | 59,1 | 22,5 | 59,6 | 22,6 |  |
| Chloride in mmol/d | 179,9 | 79,6 | 149,2*** | 60,0 | 153,7 | 65,0 | 111,7*** | 44,7 |  |
| Calcium in mmol/d | 6,2 | 3,2 | 4,3*** | 2,5 | 5,8 | 2,7 | 3,7*** | 2,1 |  |
| Magnesium in mmol/d | 4,1 | 2,0 | 4,5* | 0,1 | 3,6 | 1,6 | 3,7 | 0,1 |  |
| Phosphate in mmol/d | 29,6 | 11,5 | 31,9 | 10,2 | 24,3 | 9,0 | 22,8 | 7,1 |  |
| Urea in mmol/d | 397,3 | 144,2 | 409,0 | 123,6 | 326,2 | 118,5 | 309,8 | 86,2 |  |
| Uric acid in mmol/d | 14,9 | 4,0 | 16,4*** | 3,9 | 10,1 | 2,6 | 10,5 | 2,7 |  |

**Supplementary Table 1.** Blood and urine chemistry of recurrent calcium stone formers (stones containing > 50% of calcium) and controls according to gender. *p ≤ 0.05, ** p ≤ 0.01, *** p ≤ 0.001.
