# Supplementary material for: Serum sclerostin is associated with recurrent kidney stone formation independent of hypercalciuria
Source: Clin Kidney J. 2023 Nov 1;17(1):sfad256. doi: 10.1093/ckj/sfad256 (PMC10768761; doi:10.1093/ckj/sfad256)
Supplement: sfad256_Supplemental_Files [file sfad256_supplemental_files.zip › Supplemental table 2 correlation rKF patients_final.docx]

|  | FGF23 | age | BMI | eGFR | Ca | PO4 | PTH | 25VD | 1-25VD | Na | Cl | Bicarbonate | 24hUCa | UCa/UCrea | 24hUPO4 | UPO4/UCrea | 24hUNa | UNA/Crea |
| --- | --- | --- | --- | --- | --- | --- | --- | --- | --- | --- | --- | --- | --- | --- | --- | --- | --- | --- |
| Sclerostin | ,311*** | ,380*** | ,310*** | -,304*** | ,006 | -,051 | ,020 | ,013 | -,087 | -,142 | -,306*** | -,101 | -,054 | -,094 | ,076 | -,022 | ,094 | -,008 |
| FGF23 |  | ,325*** | ,016 | -,262** | -,104 | ,171* | ,172* | ,027 | -,157 | -,162* | -,137 | -,250** | -,237** | -,059 | -,114 | ,168* | -,203* | ,006 |
| age |  |  | ,271** | -,687*** | -,054 | -,123 | ,333*** | -,121 | -,270** | -,085 | -,065 | -,218** | -,226** | -,165* | -,001 | ,110 | -,016 | ,092 |
| BMI |  |  |  | -,272** | ,020 | -,090 | ,071 | -,062 | -,144 | -,204* | -,084 | -,273** | ,147 | ,013 | ,306*** | ,052 | ,346*** | ,061 |
| eGFR |  |  |  |  | -,060 | ,009 | -,224** | ,070 | ,249** | ,142 | ,126 | ,201* | ,267** | ,228** | ,025 | -,026 | ,024 | -,053 |
| Ca |  |  |  |  |  | ,099 | -,272** | ,103 | -,066 | ,188* | -,083 | ,270** | ,211** | ,234** | ,004 | -,047 | ,069 | -,005 |
| PO4 |  |  |  |  |  |  | -,076 | ,043 | -,011 | ,071 | -,097 | ,022 | -,004 | ,039 | ,060 | ,102 | ,037 | ,056 |
| PTH |  |  |  |  |  |  |  | -,159 | -,012 | -,064 | -,025 | -,022 | -,182* | -,207* | ,077 | ,124 | -,016 | ,042 |
| 25VD |  |  |  |  |  |  |  |  | ,421*** | -,002 | ,092 | -,153 | ,164* | ,201* | ,084 | ,146 | -,003 | ,018 |
| 1-25VD |  |  |  |  |  |  |  |  |  | ,015 | ,124 | -,035 | ,217** | ,212** | ,082 | ,058 | ,026 | -,022 |
| Na |  |  |  |  |  |  |  |  |  |  | ,400*** | ,339*** | -,011 | -,078 | ,013 | -,118 | -,078 | -,193* |
| Cl |  |  |  |  |  |  |  |  |  |  |  | -,274** | -,077 | ,000 | -,142 | -,063 | -,131 | -,007 |
| Bicarbonate |  |  |  |  |  |  |  |  |  |  |  |  | ,003 | -,054 | ,019 | -,140 | -,085 | -,219** |
| 24hUCa |  |  |  |  |  |  |  |  |  |  |  |  |  | ,792*** | ,555*** | ,366*** | ,506*** | ,239** |
| UCa/UCrea |  |  |  |  |  |  |  |  |  |  |  |  |  |  | ,171* | ,419*** | ,182* | ,341*** |
| 24hUPO4 |  |  |  |  |  |  |  |  |  |  |  |  |  |  |  | ,621** | ,599** | ,100 |
| UPO4/UCrea |  |  |  |  |  |  |  |  |  |  |  |  |  |  |  |  | ,227** | ,347*** |
| 24hUNa |  |  |  |  |  |  |  |  |  |  |  |  |  |  |  |  |  | ,649*** |
| UNA/Crea |  |  |  |  |  |  |  |  |  |  |  |  |  |  |  |  |  |  |

**Supplemental Table 2.** Spearman correlation between plasma and urine factors in rKSFs. Empty cells represent values presented elsewhere in the table. *p ≤ 0.05, **p ≤ 0.01, ***p ≤ 0.001.
