# Supplementary material for: Serum sclerostin is associated with recurrent kidney stone formation independent of hypercalciuria
Source: Clin Kidney J. 2023 Nov 1;17(1):sfad256. doi: 10.1093/ckj/sfad256 (PMC10768761; doi:10.1093/ckj/sfad256)
Supplement: sfad256_Supplemental_Files [file sfad256_supplemental_files.zip › Supplemental table 3 correlation control patients_final.docx]

|  | FGF23 | age | BMI | eGFR | Ca | PO4 | PTH | 25VD | 1-25VD | Na | Cl | Bicarbonate | 24hUCa | UCa/UCrea | 24hUPO4 | UPO4/UCrea | 24hUNa | UNA/Crea |
| --- | --- | --- | --- | --- | --- | --- | --- | --- | --- | --- | --- | --- | --- | --- | --- | --- | --- | --- |
| Sclerostin | ,104* | ,517*** | ,363*** | -,466*** | -,002 | -,123* | ,188*** | -,003 | -,003 | ,169** | -,057 | ,198*** | -,090 | -,081 | ,015 | -,032 | -,062 | -,098 |
| FGF23 |  | ,021 | ,098 | -,046 | ,268*** | ,017 | ,042 | ,132** | ,016 | ,140** | -,108* | ,151** | -,074 | -,106* | ,079 | ,074 | ,015 | ,003 |
| age |  |  | ,301*** | -,775*** | -,105* | -,157** | ,456*** | -,131** | -,023 | ,145** | ,132** | ,107* | -,068 | ,090 | -,131** | ,140** | -,042 | ,143** |
| BMI |  |  |  | -,252*** | ,061 | -,219*** | ,240*** | -,125* | -,008 | ,106* | -,010 | ,045 | ,004 | -,167** | ,291*** | -,040 | ,215*** | -,016 |
| eGFR |  |  |  |  | ,081 | ,117* | -,324*** | ,002 | ,046 | -,079 | -,090 | -,058 | ,175** | ,050 | ,151** | -,058 | ,098 | -,044 |
| Ca |  |  |  |  |  | ,008 | -,102* | -,031 | ,011 | ,301*** | -,156** | ,266*** | ,038 | ,008 | ,049 | ,043 | ,057 | ,065 |
| PO4 |  |  |  |  |  |  | -,210*** | ,123* | -,012 | ,037 | -,027 | ,000 | -,082 | ,037 | -,152*** | ,102* | -,082 | ,062 |
| PTH |  |  |  |  |  |  |  | -,386*** | -,024 | ,108* | ,142** | -,014 | -,180** | -,120** | -,063 | ,000 | -,033 | ,045 |
| 25VD |  |  |  |  |  |  |  |  | ,053 | -,041 | -,088 | ,042 | ,138** | ,182** | -,018 | ,026 | -,030 | -,011 |
| 1-25VD |  |  |  |  |  |  |  |  |  | -,091 | -,091 | ,013 | ,000 | ,003 | -,020 | ,004 | -,027 | -,056 |
| Na |  |  |  |  |  |  |  |  |  |  | ,320*** | ,254*** | ,051 | ,057 | -,008 | -,036 | ,058 | ,078 |
| Cl |  |  |  |  |  |  |  |  |  |  |  | -,283*** | ,016 | ,058 | -,026 | ,015 | ,039 | ,093 |
| Bicarbonate |  |  |  |  |  |  |  |  |  |  |  |  | ,096 | ,053 | ,053 | -,007 | -,003 | -,056 |
| 24hUCa |  |  |  |  |  |  |  |  |  |  |  |  |  | ,837*** | ,388*** | ,184*** | ,340*** | ,166*** |
| UCa/UCrea |  |  |  |  |  |  |  |  |  |  |  |  |  |  | ,009 | ,342*** | ,111* | ,306*** |
| 24hUPO4 |  |  |  |  |  |  |  |  |  |  |  |  |  |  |  | ,405*** | ,456*** | -,039 |
| UPO4/UCrea |  |  |  |  |  |  |  |  |  |  |  |  |  |  |  |  | ,034 | ,239*** |
| 24hUNa |  |  |  |  |  |  |  |  |  |  |  |  |  |  |  |  |  | ,715*** |

**Supplemental Table 3.** Spearman correlation between plasma and urine factors in control patients. Empty cells represent values presented elsewhere in the table. *p ≤ 0.05, **p ≤ 0.01, ***p ≤ 0.001.
