# Supplementary material for: Serum sclerostin is associated with recurrent kidney stone formation independent of hypercalciuria
Source: Clin Kidney J. 2023 Nov 1;17(1):sfad256. doi: 10.1093/ckj/sfad256 (PMC10768761; doi:10.1093/ckj/sfad256)
Supplement: sfad256_Supplemental_Files [file sfad256_supplemental_files.zip › Supplemental table 4.docx]

|  | **Apatite (N=17)** | **Brushite (N=5)** | **Calcium Oxalate (N=110)** | **Struvite (N=1)** | **Uric Acid (N=6)** | **Not Assessed (N=10)** |
| --- | --- | --- | --- | --- | --- | --- |
| **Sclerostin** |  |  |  |  |  |  |
| Mean (SD) | 660 (317) | 618 (219) | 748 (317) | 916 (NA) | 1010 (0.341) | 707 (290) |
| Median [Min, Max] | 678 [243, 1650] | 645 [0.342, 0.833] | 682 [287, 2210] | 916 [916, 916] | 1060 [410, 1400] | 690 [380, 1360] |

|  | **Apatite** | | **Brushite** | | **Calcium Oxalate** | | **Struvite** | **Uric Acid** | **Not Assessed** | |
| --- | --- | --- | --- | --- | --- | --- | --- | --- | --- | --- |
|  | **Female (N=13)** | **Male (N=4)** | **Female (N=1)** | **Male (N=4)** | **Female (N=25)** | **Male (N=85)** | **Female (N=1)** | **Male (N=6)** | **Female (N=5)** | **Male (N=5)** |
| **Sclerostin** |  |  |  |  |  |  |  |  |  |  |
| Mean (SD) | 710 (340) | 497 (168) | 342 (NA) | 687 (180) | 710 (263) | 759 (332) | 916 (NA) | 1010 (341) | 779 (377) | 636 (184) |
| Median [Min, Max] | 686 [243, 1650 ] | 440 [366, 743] | 342 [342, 342] | 733 [450, 833] | 652 [337, 1270] | 682 [287, 2210] | 916 [916, 916] | 1060 [410, 1400] | 749 [440, 1360] | 669 [380, 872] |

**Supplemental Table 4.** Serum sclerostin levels in pg/ml according to stone type in rKSFs.
