# Supplementary figures and images for: Serum sclerostin is associated with recurrent kidney stone formation independent of hypercalciuria
Source: Clin Kidney J. 2023 Nov 1;17(1):sfad256. doi: 10.1093/ckj/sfad256 (PMC10768761; doi:10.1093/ckj/sfad256)

## Slide 1
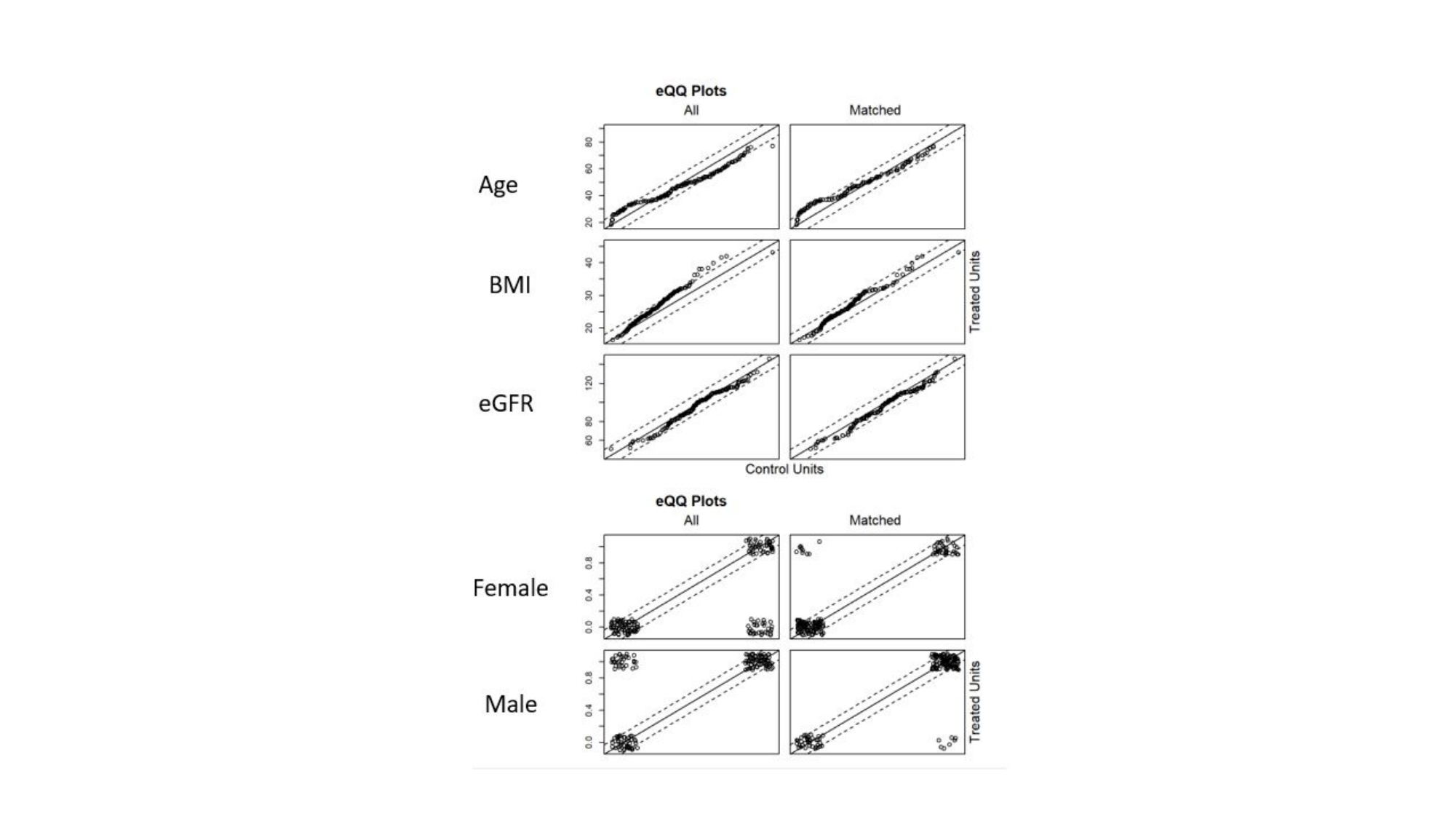

## Slide 2
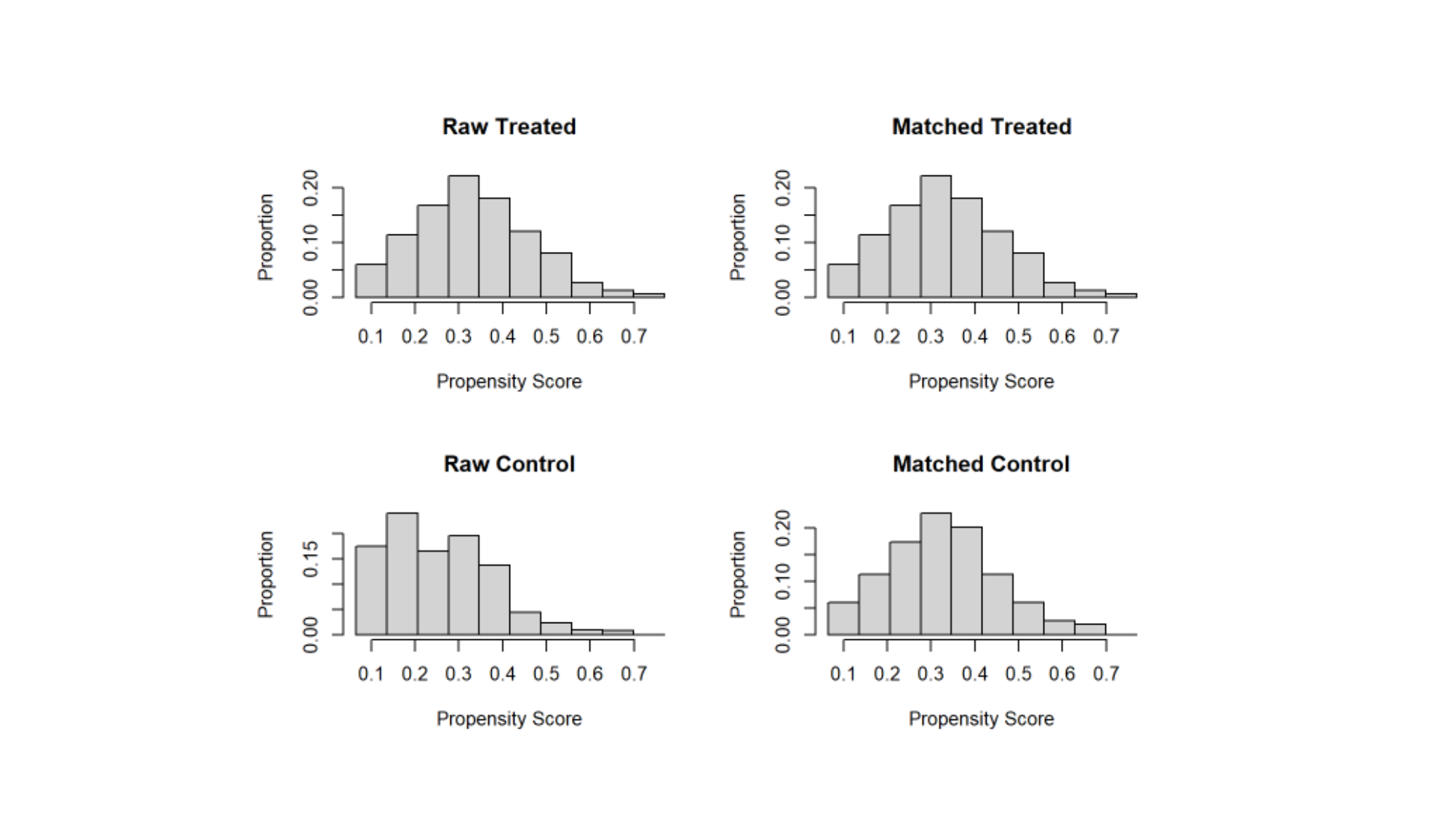

## Slide 3
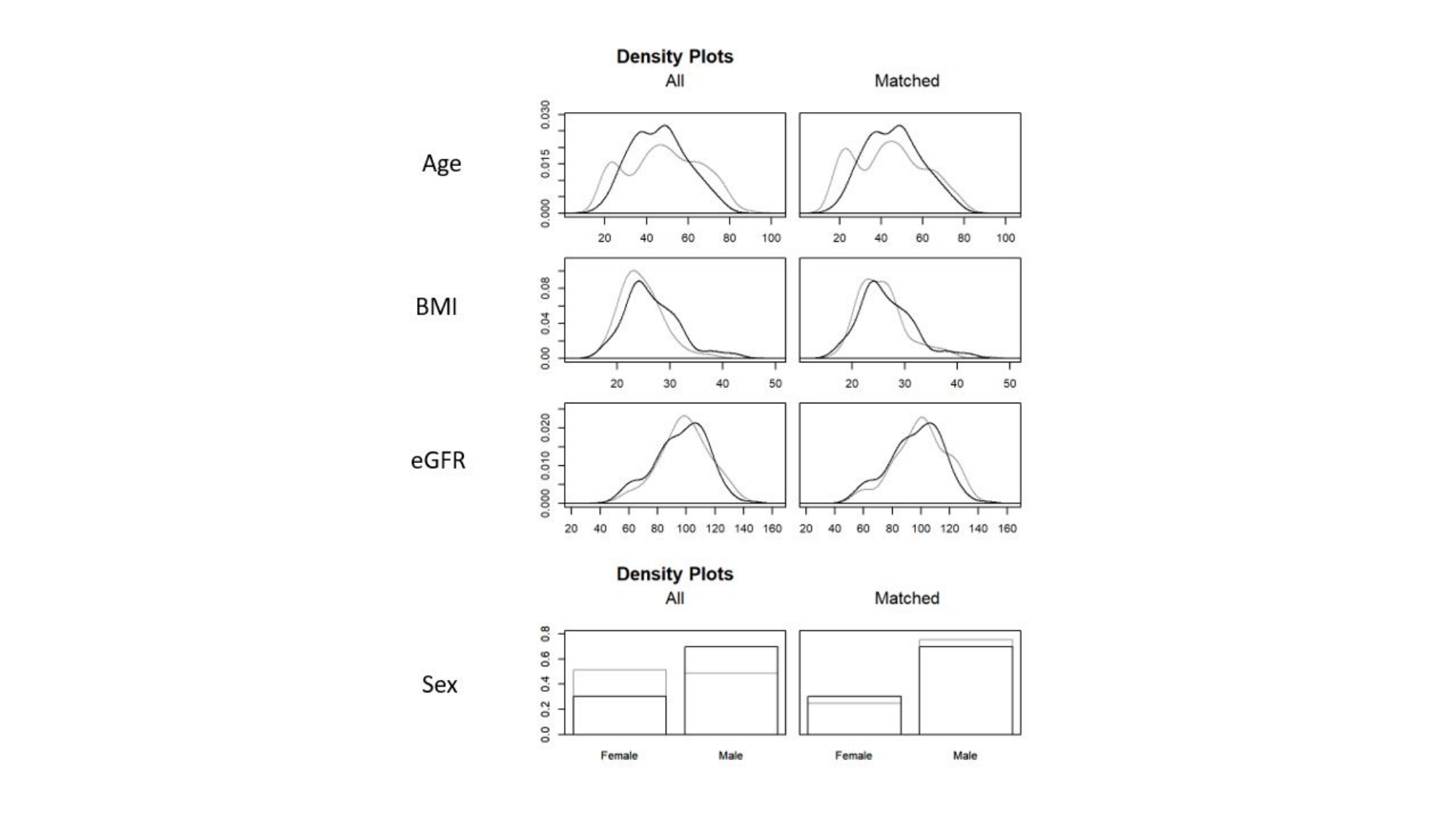

## Slide 4
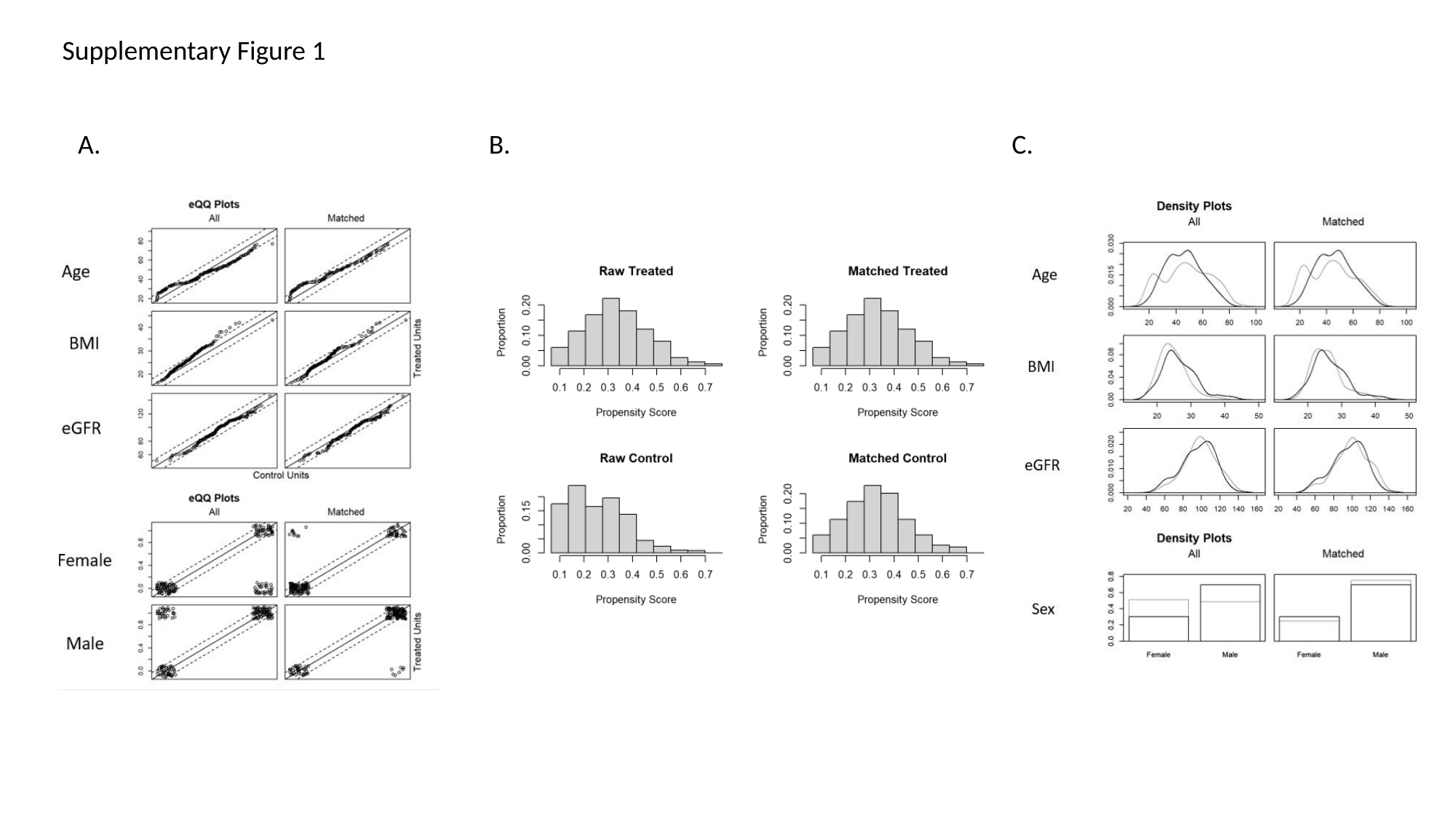

Supplementary Figure 1
A.
B.
C.

Supplement: sfad256_Supplemental_Files [file sfad256_supplemental_files.zip › Supplemental figure 1.pptx]
